# Genomic selection for productive traits in biparental cassava breeding populations

**DOI:** 10.1371/journal.pone.0220245

**Published:** 2019-07-25

**Authors:** Lívia Gomes Torres, Marcos Deon Vilela de Resende, Camila Ferreira Azevedo, Fabyano Fonseca e Silva, Eder Jorge de Oliveira

**Affiliations:** 1 Department of Plant Science, Universidade Federal de Viçosa, Viçosa, Minas Gerais, Brazil; 2 Department of Forestry Engineering, Universidade Federal de Viçosa, Viçosa, Minas Gerais, Brazil; 3 Embrapa Florestas, Colombo, Paraná, Brazil; 4 Department of Statistics, Universidade Federal de Viçosa, Viçosa, Minas Gerais, Brazil; 5 Department of Animal Science, Universidade Federal de Viçosa, Viçosa, Minas Gerais, Brazil; 6 Embrapa Mandioca e Fruticultura, Cruz das Almas, Bahia, Brazil; Shanghai Institutes for Biological Sciences, CHINA

## Abstract

Cassava improvement using traditional breeding strategies is slow due to the species’ long breeding cycle. However, the use of genomic selection can lead to a shorter breeding cycle. This study aimed to estimate genetic parameters for productive traits based on pedigree (pedigree and phenotypic information) and genomic (markers and phenotypic information) analyses using biparental crosses at different stages of selection. A total of 290 clones were genotyped and phenotyped for fresh root yield (FRY), dry matter content (DMC), dry yield (DY), fresh shoot yield (FSY) and harvest index (HI). The clones were evaluated in clonal evaluation trials (CET), preliminary yield trials (PYT), advanced yield trials (AYT) and uniform yield trials (UYT), from 2013 to 2018 in ten locations. The breeding stages were analyzed as follows: one stage (CET), two stages (CET and PYT), three stages (CET, PYT and AYT) and four stages (CET, PYT, AYT and UYT). The genomic predictions were analyzed via *k*-fold cross-validation based on the genomic best linear unbiased prediction (GBLUP) considering a model with genetic additive effects and genotype × location interactions. Genomic and pedigree accuracies were moderate to high (0.56–0.72 and 0.62–0.78, respectively) for important starch-related traits such as DY and FRY; when considering one breeding stage (CET) with the aim of early selection, the genomic accuracies ranged from 0.60 (DMC) to 0.71 (HI). Moreover, the correlations between the genomic estimation breeding values of one-stage genomic analysis and the estimated breeding values of the four-stage (full data set) pedigree analysis were high for all traits as well as for a selection index including all traits. The results indicate great possibilities for genomic selection in cassava, especially for selection early in the breeding cycle (saving time and effort).

## Introduction

Cassava (*Manihot esculenta* Crantz) is an important starchy root crop grown predominantly in the tropics that serves as a source of raw material for industrial applications and a food source for millions of people, mainly in tropical and developing regions [1,2]. Moreover, it is the world’s second most important source of starch [3], as its underground roots contain more than 80 percent starch by total dry weight [4]. Cassava is a rustic crop; it is believed to have an acclimation mechanism since it has no great environmental requirements and can grow in a wide range of climates and regions [5]. However, the average root yield is far below its potential due to its inadequate management and the use of unimproved varieties.

The use of improved varieties is one option to increase the production of the crop without increasing the planting area. Cassava breeding is based on recurrent phenotypic selection, taking advantage of vegetative propagation [6], and includes several stages of selection [7].

In general, for some traits, the improvement rate in cassava using traditional breeding strategies is slow due to the combination of several problems related to the biology of the species, such as poor flowering, a long breeding cycle, limited genetic diversity and a low multiplication rate of planting materials [8]. By virtue of the low multiplication rate of cassava, it takes several years to obtain enough planting material to conduct replicated multi-location evaluations [9]. Usually, the selection cycle requires one to two years to produce botanical seeds of the clones to be tested and four to six years of field evaluations, commonly divided into selection stages such as clonal evaluation trials, preliminary yield trials, advanced yield trials and uniform yield trials [6,10]. Selection decisions are made during this process to reduce the number of genotypes to be evaluated in replicated multi-location trials. However, traditional breeding strategies are still very demanding in terms of financial resources because cassava cultivation is highly labor-intensive and field costs can account for up to ninety percent of total costs [11].

When made possible by advancements in genotyping methods together with reduced costs per sample, information obtained at the molecular level has been used in genetic analyses to accelerate the effective selection of clones. Among molecular markers, single nucleotide polymorphisms (SNPs) stand out for broad genome coverage. SNPs occur as single nucleotide differences between individuals, and thousands of SNP markers are widely used in genome analysis [12]. According to [13], obtaining a catalog of SNPs segregating within farmers’ varieties can be used to accelerate breeding programs to improve cassava's quality, addressing nutrition and plant disease concerns.

[14] have proposed genomic selection (GS) to identify individual candidates for selection through molecular markers that are in linkage disequilibrium with loci associated with quantitative traits of interest. Thus, GS combines molecular and phenotypic data in a training population to predict the genomic estimated breeding values (GEBV) of individuals in a testing population that has only been genotyped [15]. The main advantage of genomic selection for cassava breeding programs is the ability to predict the agronomic potential of clones at an early stage and with high accuracy, thus reducing the generation interval [16]. This can accelerate the recurrent selection method [2]. Although the results are promising for some traits, we are currently in the early stages of genomic selection for cassava [10].

This study aims to (i) estimate genetic parameters (variance components, heritability and accuracy) from genomic (phenotypes and marker information) and pedigree (phenotypes and pedigree information) predictions for agronomic traits related to starch production, at different stages of a cassava breeding program; (ii) integrate clones’ breeding values into a selection index; (iii) obtain the clones’ genomic estimated breeding values through a genetic additive effect model with genotype × location interactions via Genomic Best Linear Unbiased Prediction (GBLUP); and (iv) compare the one-stage genomic analysis and the four-stage pedigree analysis to verify the feasibility of performing selection at the early stages of the breeding program using GS predictions.

## Materials and methods

### Plant material

A total of 290 cassava clones from the Cassava Breeding Program (CBP) of Embrapa Mandioca e Fruticultura (Cruz das Almas, Brazil) were genotyped and phenotyped for some important agronomic traits. The set of clones consisted of populations of full- and half-siblings obtained from 30 biparental crosses; commercial clones were used as parents and control trials.

### Experimental design and phenotypical evaluation

Cassava clones were evaluated at different selection stages: clonal evaluation trials (CET), preliminary yield trials (PYT), advanced yield trials (AYT) and uniform yield trials (UYT), in field trials carried out from 2013 to 2018 in ten locations within Bahia State that included Cruz das Almas, Santo Amaro and Laje (Table 1).

**Table 1:** Experimental areas, cities and geographical coordinates.

Experimental Area	City	Geographical Coordinates
CNPMF	Cruz das Almas	12°40'42.4"S 39°05'27.8"W
Capela	Laje	13°10'37.8"S 39°14'22.4"W
Novo Horizonte	Laje	13°06'38.4"S 39°16'20.4"W
Novo Rumo	Laje	13°15'33.5"S 39°14'12.8"W
Rio de Areia1	Laje	13°08'51.1"S 39°17'59.7"W
Rio de Areia2	Laje	13°07'37.5"S 39°19'02.1"W
Santo Amaro	Santo Amaro	12°32'58.0"S 38°48'16.1"W
São Jorge	Laje	13°07'01.3"S 39°18'08.6"W
Sombra Verde	Laje	13°13'08.4"S 39°19'27.8"W
UFRB	Cruz das Almas	12°39'14.3"S 39°04'49.0"W

The CET plots each consisted of a single row with 5–8 plants per plot, in an augmented block design of 6–19 blocks, with 3–11 checks as a control (with the exception of one field trial that was a complete randomized block design with 2 reps). PYT plots contained two rows with 20 plants per plot, in a complete randomized block design with 2 reps and 4–8 checks as a control. AYT plots contained four rows with 36 plants per plot, in a complete randomized block design with 3 reps and 3–5 checks as a control. Finally, UYT plots included four rows with 80 plants per plot, in a complete randomized block design with 3 reps and 3–6 checks as a control.

Planting was performed from May to July (during the rainy season). Spacings between rows and plants were 0.90 and 0.80 meters, respectively. All trait management was performed, whenever necessary, in accordance with the technical recommendations and standard agricultural practices for cassava [17]. Plants were harvested 11–12 months after planting. All plants in the plots were evaluated.

The traits evaluated were as follows: fresh root yield (FRY, in t ha^-1^), dry matter content by specific gravity method (DMC, %), measured using the gravimetric method described by [18] and expressed in %; dry yield (DY, in t ha^-1^), determined by multiplying FRY by DMC; fresh shoot yield (FSY, in t ha^-1^) and harvest index (HI, %). HI is the proportion of fresh root weight in total biomass.

### Genotyping and data quality control

The genotyping of the 290 clones was performed at Cornell University. The DNA was extracted from the leaves of cassava accessions using cetyltrimethylammonium bromide according to the protocol of [19]. Then, the DNA concentration was adjusted to 60 ng/μl, and the samples were sent to the Genomic Diversity laboratory for library preparation, sequencing and bioinformatics analyses. The genotyping by sequencing (GBS) protocol is described in detail by [20]. The DNA was digested with an ApeKI enzyme, a type II restriction endonuclease that recognizes a degenerate five-base sequence and creates a 5’ overhang (3 bp). The linkage between ApeKI-cut genomic DNA and adapter was made after the digestion of the samples and the 192-multiplex samples for sequencing. The *Genome Analyser 2000* (Illumina, Inc., San Diego, CA, USA) was used to perform the GBS.

Data quality control was performed for markers; indels and markers with minor allele frequency (MAF) less than 0.0415—calculated by the formula $\frac{1}{\sqrt{2N}}$, where N is the number of clones [21]—and a call rate of less than 0.90 were removed from the analyses. The GBS coverage was approx. 72%. Excluding the indels, the dataset consisted of 209,316 SNP markers. After quality control, the final dataset used to perform the genomic analyses consisted of 51,259 SNPs, with mean heterozygosity of 0.10 and mean missingness of 3.34%. The missing data were imputed by the heterozygote [22].

### Pedigree and genomic statistical analyses

Pedigree and genomic analyses were performed. The pedigree analyses were based on the pedigree information, whereas for the genomic analyses, a genomic kinship matrix was constructed based on the information provided by the 51,259 SNP markers. The clones were evaluated at the four selection stages (CET, PYT, AYT and UYT) for FRY, DMC, DY, FSY and HI. Statistical analyses were performed by splitting the dataset by the stages of the breeding program and analyzing subsets of data with different numbers of stages. The breeding stages were analyzed as follows: one stage (CET), two stages (CET and PYT), three stages (CET, PYT and AYT) and four stages (CET, PYT, AYT and UYT) (Table 2). This division was made to assess the differences in the genetic parameters that appeared with the addition of new trials and therefore new combinations of year, location and block within location, taking into consideration the unbalance common in this type of system. The overall aim was to verify the possibility of performing selection at an earlier stage of a breeding program (CET).

**Table 2:** (A) Selection stages, locations, years, number of observations and number of clones; and (B) Datasets established by the number of stages included in the analysis, locations, years, number of observations and number of clones.

(A)				
Stages	Locations	Years	Number of observations	Number of clones
Clonal evaluation trials (CET)	CNPMF, Novo Horizonte, Novo Rumo and RiodeAreia1	2013, 2014, 2015, 2016 and 2018	964	290
Preliminary yield trials (PYT)	Capela and Novo Horizonte	2014 and 2017	288	136
Advanced yield trials (AYT)	Novo Horizonte, RiodeAreia1, Santo Amaro and UFRB	2015, 2016 and 2018	388	72
Uniform yield trials (UYT)	Novo Horizonte, RiodeAreia1, RiodeAreia2, Santo Amaro, São Jorge, Sombra Verde and UFRB	2016, 2017 and 2018	315	21
(B)				
1 stage: CET	CNPMF, Novo Horizonte, Novo Rumo and RiodeAreia1	2013, 2014, 2015, 2016 and 2018	964	290
2 stages: CET and PYT	Capela, CNPMF, Novo Horizonte, Novo Rumo and RiodeAreia1	2013, 2014, 2015, 2016, 2017 and 2018	1252	290
3 stages: CET, PYT and AYT	Capela, CNPMF, Novo Horizonte, Novo Rumo, RiodeAreia1, Santo Amaro and UFRB	2013, 2014, 2015, 2016, 2017 and 2018	1640	290
4 stages: CET, PYT, AYT and UYT	Capela, CNPMF, Novo Horizonte, Novo Rumo, RiodeAreia1, RiodeAreia2, Santo Amaro, São Jorge, Sombra Verde and UFRB	2013, 2014, 2015, 2016, 2017 and 2018	1955	290

The models used for the pedigree (P) and genomic analyses through markers (M) are as follows, respectively:

$$y=Xb+Z_{1}a_{P}+Z_{2}a_{LP}+\varepsilon$$ (1)

$$y=Xb+Z_{1}a_{M}+Z_{2}a_{LM}+\varepsilon$$ (2)

where **y** is the vector of phenotypic observations and ***b*** is the vector of fixed effects considering the combination of year, location and block within location added to the mean. The vectors ***a***_***P***_ and ***a***_***M***_ refer to the random additive genotype effects, ***a***_***P***_ ~ N(**0**, $\sigma_{aP}^{2}A$) in the pedigree analysis and ***a***_***M***_~ N(**0**, $\sigma_{aM}^{2}G$) in the genomic analysis, where **A** is the pedigree relationship matrix, **G** is the additive genomic relationship matrix, $\sigma_{aP}^{2}$ is the genetic variance estimate associated with ***a***_***P***_ and $\sigma_{aM}^{2}$ is the genetic variance associated with ***a***_***M***_. The random effect vectors ***a***_***LP***_ and ***a***_***LM***_ refer to the genotype × location interaction effect, ***a***_***LP***_ ~ N(**0**, $\sigma_{alP}^{2}A_{L}$) in the pedigree analysis, and ***a***_***LM***_ ~ N(**0**, $\sigma_{alM}^{2}G_{L}$) in the genomic analysis, where **A**_***L***_ is the block-diagonal of each location pedigree relationship matrix, **G**_***L***_ is the block-diagonal of each location genomic relationship matrix, $\sigma_{alP}^{2}$ is the variance estimate due to genotype × location interactions with phenotype and $\sigma_{alM}^{2}$ is the variance estimate due to genotype × location interactions with marker information. **X**, **Z**_**1**_ and **Z**_**2**_ are the incidence matrices for fixed effects and the random effects of genotypes and genotype × location interactions, respectively. **ε** represents the residual vector (**ε** ~ N(**0**, $\sigma_{e}^{2}I$)), where $\sigma_{e}^{2}$ is the residual variance estimate. We estimated heritability as the ratio of the genetic variance to the sum of the genetic variance, the variance due to the genotype × location interaction and the variance of the residuals (total variance) in each model; the interaction’s coefficient of determination was estimated by the ratio of the variance due to the genotype × location interaction and the total variance.

The mixed model equations for best linear unbiased prediction (BLUP) (1) are as follows:

$$\left[\begin{array}{ccc} X\prime X & X\prime Z_{1} & X\prime Z_{2}\\ Z_{1}\prime X & Z_{1}\prime Z_{1}+A^{-1}\frac{\sigma_{e}^{2}}{\sigma_{aP}^{2}} & Z_{1}\prime Z_{2}\\ Z_{2}\prime X & Z_{2}\prime Z_{1} & Z_{2}^{\prime }Z_{2}+A_{L}^{-1}\frac{\sigma_{e}^{2}}{\sigma_{alP}^{2}} \end{array}\right]\left[\begin{array}{c} \hat{b}\\ {\hat{a}}_{P}\\ {\hat{a}}_{LP} \end{array}\right]=\left[\begin{array}{c} X\prime y\\ Z_{1}\prime y\\ Z_{2}\prime y \end{array}\right]$$

where **A** is the pedigree relationship matrix and **A**_***L***_ is the block-diagonal of **An**_**i**_ matrices of pedigree relationship matrix in each location:

$$A_{L}=\left[\begin{array}{cccc} A_{1} & 0 & 0 & 0\\ 0 & A_{2} & 0 & 0\\ 0 & 0 & \ddots & \vdots \\ 0 & 0 & \ldots & {An}_{i} \end{array}\right]$$

where n_i_ = [4, 5, 7, 10] is the number of locations and i = [1, 2, 3, 4] is the number of stages of evaluation.

The size of **A** is 290 × 290, regardless of the number of stages included in the analysis. The size of the **A**_**L**_ matrix is 457 × 457, 581 × 581, 698 × 698 and 757 × 757 when one, two, three and four stages are included in the analysis, respectively.

The equations of mixed genomic best linear unbiased prediction (GBLUP) models (2) [23] are given as follows:

$$\left[\begin{array}{ccc} X\prime X & X\prime Z_{1} & X\prime Z_{2}\\ Z_{1}\prime X & Z_{1}\prime Z_{1}+G^{-1}\frac{\sigma_{e}^{2}}{\sigma_{aM}^{2}} & Z_{1}\prime Z_{2}\\ Z_{2}\prime X & Z_{2}\prime Z_{1} & Z_{2}^{\prime }Z_{2}+G_{L}^{-1}\frac{\sigma_{e}^{2}}{\sigma_{alM}^{2}} \end{array}\right]\left[\begin{array}{c} \hat{b}\\ {\hat{a}}_{M}\\ {\hat{a}}_{LM} \end{array}\right]=\left[\begin{array}{c} X\prime y\\ Z_{1}\prime y\\ Z_{2}\prime y \end{array}\right]$$

where $G=\frac{MM\prime }{\sum_{i=1}^{n}2p_{i}q_{i}}$ and **G** is the genomic relationship matrix, p_i_ and q_i_ are the allele frequencies of marker i, and **M** is the markers incidence matrix centered by the mean 2p_i_. **G**_***L***_ is a block-diagonal matrix of **Gn**_**i**_ matrices in each location:

$$G_{L}=\left[\begin{array}{cccc} G_{1} & 0 & 0 & 0\\ 0 & G_{2} & 0 & 0\\ 0 & 0 & \ddots & \vdots \\ 0 & 0 & \ldots & {Gn}_{i} \end{array}\right]$$

where n_i_ = [4, 5, 7, 10] is the number of locations and i = [1, 2, 3, 4] is the number of stages of evaluation.

The size of **G** is 290 × 290 regardless of the number of stages included in the analysis. The size of the **G**_**L**_ matrix is 457 × 457, 581 × 581, 698 × 698 and 757 × 757 when one, two, three and four stages are included in the analysis, respectively.

The SNP effects ($\hat{m}$) are calculated as follows:

$$\hat{m}={\left(MM\prime \right)}^{-1}M^{\prime }{\hat{a}}_{M}$$

The pedigree and genomic analyses were performed using the methodology of mixed models via REML/BLUP, which combines the genetic values of BLUP (best non-biased linear prediction; in genomic analysis it is called GBLUP because it depends on the G matrix) prediction procedure with the REML (residual or restricted maximum likelihood) estimation of variance components. A Likelihood Ratio Test (LRT) and deviance analysis were undertaken to test the random effects, which were tested using the chi-square statistic [24].

The cross-validation method used was the ten-*fold*, and the clones were randomly assigned to each fold. The training set, composed of 9 of the 10 subsets, was used to estimate marker effects and the remaining subset was the validation set. These marker effects estimates were used to predict the genomic breeding values of the validation set individuals. This process was repeated until all 10 subsets had served as the validation population once. Trait estimates of predictive ability, accuracy and bias were calculated from cross-validation with the training set for pedigree and genomic analyses. The selection efficiency measures were estimated at each fold, and the value presented in this study is the mean of the folds.

The predictive ability was given by the correlation coefficient between predicted genetic values (PGVs) and predicted genetic values added to the mean of the residuals in the validation population. PGVs were the estimated breeding values (EBVs) for the pedigree analysis and the genomic estimated breeding values (GEBVs) for the genomic analysis. Predicted genetic values added to the mean of the residuals for each clone can be interpreted as pseudo-phenotypes (PPs). We adopted this type of cross-validation due to the considerable imbalance of the dataset. For example, the control clones (some of which were parents of other clones to be tested) were replicated at several locations and through the years, whereas many other clones to be tested were replicated only a few times (due to the limited planting material) (S1 Dataset).

The accuracy, one of the main measures to compare models and methods in genomic selection, was calculated as the ratio between the predictive ability and the square root of the phenotypic trait heritability. This measure indicates how accurate the model is in estimating genetic values.

The PPs were linearly regressed on the PGVs, and the regression coefficient ${\hat{b}}_{\mathrm{PP},\mathrm{PGV}}$ was used to measure the degree of bias of the PGV prediction. The bias relates to the size of the absolute differences between clones’ predicted genetic values and their pseudo-phenotypes. The estimated magnitude of these differences can be quantified by the ${\hat{b}}_{\mathrm{PP},\mathrm{PGV}}$ regression coefficient and can be overestimated (${\hat{b}}_{\mathrm{PP},\mathrm{PGV}}$ < 1) or underestimated (${\hat{b}}_{\mathrm{PP},\mathrm{PGV}}$ > 1). A regression coefficient equal to one indicates no bias. Then, here we will represent bias as one unit minus the regression coefficient ${\hat{b}}_{\mathrm{PP},\mathrm{PGV}}$ (Bias = $1‐{\hat{b}}_{\mathrm{PP},\mathrm{PGV}}$). Statistical analyses were performed using the package *sommer* [25] in the software R [26].

### Selection index

After obtaining the EBVs and GEBVs of the clones, we calculated an empirical selection index (SI) that integrates five relevant traits, assigning them weights based on the breeding program’s objective:

SI = (FRY*10) + (DMC*10) + (DY*10) + (FSY*5) + (HI*3) (S1 Dataset)

## Results

### Pedigree analyses

The set of clones presented genetic variability for all evaluated traits (*p* ≤ 0.01). In relation to the genotype × location interaction effect (GxL), when only one stage was evaluated, the clones presented different behaviors in relation to environmental changes for FRY, DY and FSY (*p* ≤ 0.01); however, when four stages were included in the analysis, the additive GxL interaction effect was significant (*p* ≤ 0.01) for all of the traits (Table 3).

**Table 3:** Pedigree analyses: Comparison of models by Likelihood Ratio Test (LRT).

One stage					
LRT ^/3^					
Model	FRY	DMC	DY	FSY	HI
Complete model x Restricted model (without G^/1^)	81.71**	144.46**	78.61**	61.00**	77.45**
Complete model x Restricted model (without GxL^/2^)	14.46**	0.00	13.83**	51.03**	-0.25
Four stages					
LRT					
Model	FRY	DMC	DY	FSY	HI
Complete model x Restricted model (without G)	160.18**	306.91**	148.09**	155.56**	180.85**
Complete model x Restricted model (without GxL)	38.20**	11.08**	38.33**	132.03**	39.68**

For each trait, three models were compared based on the Likelihood Ratio Test (LRT)

^/1^ G: genotype effects

^/2^ GxL: genotype by location interaction effects

^/3^ LRT = 2*(complete model log-likelihood—restricted model log-likelihood), ** significant at 0.01 and * significant at 0.05 by the likelihood ratio test.

Trait heritabilities ranged from 0.42 to 0.73, i.e., from moderate to high. DMC and HI were the traits with the highest heritabilities (ranging from 0.69 to 0.73 and from 0.56 to 0.60, respectively), while heritability of the other traits varied across the analyzed data sets (Table 4). The variation of the genetic parameters with the increment of selection stages occurs due to the addition of phenotypic observations, and therefore information that comes from different experimental designs. Successive stages of selection gradually reduce the number of genotypes as plot-size and locations increase [6]. Trait heritabilities were higher when the full data set (four-stage) was analyzed, with values of 0.52, 0.50, 0.52 and 0.60 for FRY, DY, FSY and HI, respectively, with the exception of DMC, which exhibited higher heritability when one stage was analyzed (0.73). DMC and FSY were the traits with the lowest and highest coefficients of determination of the interaction, respectively. The pedigree means varied from 29.24–31.81 t ha^-1^, 33.41–33.54%, 9.63–10.61 t ha^-1^, 12.25–13.65 t ha^-1^ and 70.62–73.86% for FRY, DMC, DY, FSY and HI, respectively, depending on the number of stages included in the analysis.

**Table 4:** Estimates of heritability, mean, coefficient of variation (CVe), predictive ability, accuracy and bias for pedigree analyses.

	One stage						
Traits^/1^	Heritability	Interaction’s Coefficient of Determination	Mean	CVe	Predictive Ability	Accuracy	Bias
FRY	0.49	0.07	29.24	0.36	0.44±0.18	0.63±0.25	-0.03±0.51
DMC	0.73	0.00	33.49	0.05	0.54±0.09	0.64±0.11	0.01±0.27
DY	0.47	0.07	9.63	0.39	0.42±0.19	0.62±0.27	-0.03±0.51
FSY	0.48	0.12	12.25	0.82	0.45±0.15	0.64±0.22	0.01±0.40
HI	0.58	0.00	70.62	0.13	0.52±0.22	0.69±0.29	0.08±0.34
	Two stages						
FRY	0.44	0.08	31.34	0.31	0.51±0.12	0.76±0.18	-0.01±0.21
DMC	0.70	0.00	33.48	0.05	0.61±0.08	0.73±0.10	-0.03±0.17
DY	0.42	0.08	10.53	0.33	0.51±0.13	0.78±0.20	-0.03±0.22
FSY	0.42	0.17	13.65	0.67	0.45±0.17	0.70±0.26	0.05±0.34
HI	0.56	0.06	72.84	0.11	0.50±0.16	0.67±0.21	0.09±0.31
	Three stages						
FRY	0.46	0.08	31.81	0.29	0.49±0.15	0.72±0.23	0.07±0.27
DMC	0.69	0.01	33.41	0.04	0.63±0.08	0.75±0.10	-0.06±0.11
DY	0.45	0.09	10.61	0.31	0.50±0.15	0.74±0.22	0.05±0.26
FSY	0.42	0.19	12.51	0.68	0.46±0.16	0.70±0.24	0.07±0.40
HI	0.58	0.06	73.86	0.11	0.53±0.13	0.69±0.17	0.08±0.27
	Four stages						
FRY	0.52	0.08	31.36	0.27	0.53±0.13	0.73±0.18	0.02±0.23
DMC	0.70	0.04	33.54	0.04	0.61±0.10	0.73±0.12	-0.02±0.12
DY	0.50	0.09	10.48	0.29	0.53±0.13	0.75±0.19	0.00±0.23
FSY	0.52	0.17	12.66	0.62	0.55±0.13	0.77±0.19	-0.02±0.33
HI	0.60	0.07	73.86	0.10	0.53±0.12	0.69±0.16	0.08±0.22

^/1^ FRY–fresh root yield (t ha^-1^); DMC–dry matter content (%); DY–dry yield (t ha^-1^); FSY–fresh shoot yield (t ha^-1^); and HI–harvest index (%).

The coefficient of residual variation (CVe), which is a measure of dispersion and relative variability, ranged from 0.04 to 0.82 across traits and datasets (number of stages included), being higher for FSY (0.62–0.82) and lower for DMC (0.04–0.05). In general, for all traits, the CVe decreased when a greater number of stages was included in the analysis.

Trait predictive abilities ranged from 0.42–0.54, 0.45–0.61, 0.46–0.63 and 0.53–0.61 when one, two, three and four stages were included in the analyses, respectively. For FRY, the predictive ability was 0.44, 0.51, 0.49 and 0.53, when one, two, three and four stages were included in the analyses. For DY, the predictive ability also increased continuously (except for a small reduction in the three-stage analysis), with values of 0.42, 0.51, 0.50 and 0.53 when one, two, three and four stages were included in the analyses. That increasing pattern occurred for all traits with the exception of HI, for which the predictive ability did not alter much, ranging from 0.50 to 0.53.

Trait accuracies ranged from 0.62–0.69, 0.67–0.78, 0.69–0.75 and 0.69–0.77 when one, two, three and four stages were included in the analyses, respectively (Table 4). FRY, DMC, DY and FSY presented accuracies greater than 0.70 when more than one stage was included in the analyses (two-, three- and four-stage datasets). Accuracies above 0.70 are very suitable from the selection practice point of view. According to [27], accuracy values between 0.70 and 0.90 are classified as high precision and values above 0.90 as very high precision. The increase in accuracy and predictive ability may reflect a greater experimental reliability of the stages included in the analyses. When we think of the one-stage analysis, with CET only, this field trial was performed with single rows with 6–8 plants per plot, differing from the subsequent stages, which included replications and were performed with 2–4 rows and a greater number of plants per plot. FRY and DY presented higher accuracies when four stages were included in the analysis compared to when only one was evaluated. For FRY, these values were 0.63 and 0.73, and for DY, they were 0.62 and 0.75. For the other traits, those differences in accuracy were of approximately the same amplitude, except for HI, for which the accuracy was 0.69 whether one or four stages were included in the analyses.

The bias ranged from -0.06 (DMC, three stages) to 0.09 (HI, two stages), with the lowest amplitude (highest bias value minus lowest bias value) occurring when four stages were evaluated. In general, the traits presented little bias, ranging from -0.03 to 0.07 for FRY, from -0.06 to 0.01 for DMC, from -0.03 to 0.05 for DY, from -0.02 to 0.07 for FSY and from 0.08 to 0.09 for HI. DY presented zero bias when four stages were included in the analysis. HI was the trait that presented the highest values of bias; therefore, the estimates of the genetic values for HI were slightly overestimated.

Indirect selection is an option when there is a linear relationship between two traits and one trait either has low heritability or is difficult to measure compared to the other trait. For example, FSY is relatively easier to measure than root-related traits, and it had moderate positive correlations with FRY (0.41–0.48) and DY (0.43–0.49) and negative and moderate to high correlations with HI ((-0.59)–(-0.62)). FRY was highly and positively correlated with DY (0.99) (Table 5). HI was negatively correlated with DMC (-0.31 and -0.19 in the one-stage and four-stage analyses, respectively). The correlations between vectors of estimated breeding values obtained through the different analyses (with a different number of stages of selection—one and four stages) are presented in the diagonal of Table 5. The high correlations, ranging from 0.84 to 0.88, indicate good correspondence between the one-stage and four-stage EBVs, which might allow early selection.

**Table 5:** Trait correlation estimates between vectors of EBVs from pedigree analysis, above the diagonal considering the four stages of evaluation, and below the diagonal considering only one stage of evaluation; along the diagonal is the correlation between vectors of genetic values through the different analyses of stages of selection.

Traits^/1^	FRY^/2^	DMC	DY	FSY	HI
FRY	0.88**	-0.04^ns^	0.99**	0.48**	0.26**
DMC	-0.10^ns^	0.84**	0.11^ns^	0.09^ns^	-0.19**
DY	0.99**	0.04^ns^	0.88**	0.49**	0.24**
FSY	0.41**	0.17**	0.43**	0.87**	-0.62**
HI	0.35**	-0.31**	0.32**	-0.59**	0.87**

^/1^ FRY–fresh root yield (t ha^-1^); DMC–dry matter content (%); DY–dry yield (t ha^-1^); FSY–fresh shoot yield (t ha^-1^); and HI–harvest index (%)

^2/^** Significant at *p* ≤ 0.01, * Significant at *p* ≤ 0.05, and ^ns^ not significant.

### Genomic analyses

The set of clones presented genetic variability for all evaluated traits (*p ≤* 0.01), as was shown for the pedigree analyses. The GxL interaction effect was significant (*p ≤* 0.01) for all traits with the exception of DMC (Table 6).

**Table 6:** Genomic analyses: comparison of models by Likelihood Ratio Test (LRT).

One stage					
LRT ^/3^					
Model	FRY	DMC	DY	FSY	HI
Complete model x Restricted model (without G^/1^)	108.60**	159.53**	106.11**	96.91**	105.42**
Complete model x Restricted model (without GxL^/2^)	18.38**	0.00	19.09**	47.19**	6.65**
Four stages					
LRT					
Model	FRY	DMC	DY	FSY	HI
Complete model x Restricted model (without G)	151.58**	381.14**	149.84**	194.97**	315.53**
Complete model x Restricted model (without GxL)	60.64**	1.55	61.52**	139.10**	19.85**

For each trait, three models were compared based on the Likelihood Ratio Test (LRT)

^/1^ G: genotype effects

^/2^ GxL: genotype by location interaction effects

^/3^ LRT = 2*(complete model log-likelihood—restricted model log-likelihood), ** significant at *p* ≤ 0.01 and * significant at *p* ≤ 0.05 by the likelihood ratio test.

The traits presented moderate to high heritability, ranging from 0.38–0.48, 0.66–0.69, 0.36–0.46, 0.41–0.51 and 0.47–0.60 for FRY, DMC, DY, FSY and HI, respectively (Table 7). In general, heritability estimates obtained in the genomic analyses were lower than those obtained by the pedigree analyses. However, the differences in heritabilities estimated through pedigree and genomic analyses were smaller when a larger number of stages of selection was included in the analysis. DMC was the trait that presented the highest heritabilities, whereas DY presented the lowest heritabilities, regardless of the dataset evaluated. As was found in the pedigree analyses, in the genomic analyses DMC and FSY were the traits with the lowest and highest coefficients of determination of the interaction, respectively.

**Table 7:** Estimates of heritability, mean, coefficient of variation (CVe), predictive ability, accuracy and bias for genomic analyses.

	One stage						
Traits^/1^	Heritability	Interaction’s Coefficient of Determination	Mean	CVe	Predictive Ability	Accuracy	Bias
FRY	0.39	0.06	29.67	0.36	0.43±0.13	0.61±0.19	0.04±0.40
DMC	0.66	0.00	33.43	0.05	0.51±0.09	0.60±0.10	0.05±0.27
DY	0.36	0.06	9.87	0.38	0.43±0.14	0.63±0.20	0.03±0.40
FSY	0.45	0.09	14.15	0.71	0.48±0.11	0.69±0.17	-0.02±0.30
HI	0.47	0.04	72.76	0.13	0.54±0.20	0.71±0.26	0.01±0.28
	Two stages						
FRY	0.38	0.08	29.61	0.33	0.46±0.11	0.69±0.16	0.06±0.23
DMC	0.67	0.00	33.42	0.05	0.57±0.12	0.68±0.14	0.00±0.22
DY	0.37	0.08	9.85	0.35	0.47±0.11	0.72±0.17	0.01±0.21
FSY	0.41	0.13	15.80	0.58	0.44±0.21	0.68±0.32	0.11±0.40
HI	0.49	0.06	70.19	0.12	0.42±0.21	0.56±0.28	0.27±0.38
	Three stages						
FRY	0.42	0.08	29.15	0.32	0.44±0.14	0.65±0.21	0.10±0.28
DMC	0.68	0.00	33.22	0.05	0.58±0.10	0.70±0.12	-0.02±0.15
DY	0.39	0.08	9.65	0.34	0.45±0.13	0.67±0.20	0.07±0.24
FSY	0.43	0.12	12.47	0.70	0.47±0.17	0.72±0.26	0.10±0.34
HI	0.56	0.03	73.74	0.11	0.47±0.19	0.62±0.25	0.22±0.33
	Four stages						
FRY	0.48	0.08	31.17	0.27	0.50±0.12	0.69±0.17	0.03±0.24
DMC	0.69	0.01	33.23	0.04	0.59±0.10	0.70±0.12	-0.01±0.14
DY	0.46	0.08	10.37	0.29	0.51±0.11	0.72±0.16	-0.01±0.21
FSY	0.51	0.12	14.18	0.56	0.50±0.16	0.70±0.23	0.07±0.29
HI	0.60	0.03	73.58	0.11	0.48±0.17	0.61±0.22	0.23±0.28

^/1^ FRY–fresh root yield (t ha^-1^); DMC–dry matter content (%); DY–dry yield (t ha^-1^); FSY–fresh shoot yield (t ha^-1^); and HI–harvest index (%).

In general, the predictive ability was higher when four stages were included in the analysis, except for HI, for which it decreased. For FRY, DMC and DY, the predictive abilities were higher in the four-stage analyses (0.50, 0.59 and 0.51, respectively) and lower in the one-stage analyses (0.43, 0.51 and 0.43, respectively); for FSY, the predictive ability was higher in the four-stage analysis (0.50) but lower in the two-stages analysis (0.44) compared to one stage. For HI, predictive ability was also higher for the one-stage analysis (0.54) than for the two-stage analysis (0.42).

The accuracies ranged from 0.61–0.69, 0.60–0.70, 0.63–0.72, 0.68–0.72 and 0.56–0.71 for FRY, DMC, DY, FSY and HI, respectively (Table 7). Across the analyses based on different datasets (with a different number of stages included), accuracy estimates varied (but they were at least greater than 0.56). As an example, for DY, the accuracy values were 0.63, 0.72, 0.67 and 0.72 when one, two, three and four stages were included in the analyses. For HI, the scenario was quite different, with a large reduction in accuracy from one to two stages, with values of 0.71, 0.56, 0.62 and 0.61 when one, two, three and four stages were included. The inclusion of more information and the imbalance common for this type of system (more stages–selection included–fewer clones–more locations in the latter stages) may have affected the traits differently in relation to parameters of genomic selection precision.

In general, the traits presented no or very little bias, with the exception of HI when two, three or four stages were included in the analyses. As shown in the pedigree analysis, the genetic values for HI were overestimated. The bias in the genomic analyses varied slightly more, in terms of amplitude, when compared to the biases from the pedigree analyses (they ranged from -0.02 to 0.27 for genomic analyses and from -0.06 to 0.09 for pedigree analyses).

The estimates of the correlations based on the GEBVs (Table 8) were similar to the estimates of the correlations based on the EBVs (Table 5). The most expressive differences of trait correlations presented in Tables 5 and 8 were between the trait pairs FRY and FSY, FRY and HI, DY and FSY, DY and HI, and DMC and HI. FRY and DY were highly and positively correlated (0.99 with one-stage and four-stage datasets). HI and FSY were negatively correlated, with values of -0.62 and -0.71 when one and four stages were included in the analyses, respectively. The differences in correlation with respect to the stage of the breeding program may be explained by the fact that the relationships among traits change through the breeding program due to selection [6]. The diagonal of Table 8 shows the correlation between vectors of GEBVs through the different analyses (with different numbers of stages of selection—one and four stages). These values (ranging from 0.80 to 0.86) indicate good correspondence between the vectors of GEBVs from one- and four-stage analyses, which may be evidence in favor of early selection.

**Table 8:** Trait correlation estimates between vectors of GEBVs from genomic analysis, above the diagonal considering the four stages of evaluation, and below the diagonal considering only one stage of evaluation; along the diagonal is the correlation between vectors of genomic values through the different analyses of stages of selection.

Traits^/1^	FRY^/2^	DMC	DY	FSY	HI
FRY	0.81**	-0.05^ns^	0.99**	0.40**	0.37**
DMC	-0.05^ns^	0.86**	0.11^ns^	0.11^ns^	-0.20**
DY	0.99**	0.07^ns^	0.80**	0.41**	0.34**
FSY	0.35**	0.10^ns^	0.35**	0.86**	-0.61**
HI	0.41**	-0.21**	0.40**	-0.62**	0.85**

^/1^ FRY–fresh root yield (t ha^-1^); DMC–dry matter content (%); DY–dry yield (t ha^-1^); FSY–fresh shoot yield (t ha^-1^); and HI–harvest index (%)

^2/^** Significant at *p* ≤ 0.01, * Significant at *p* ≤ 0.05, and ^ns^ not significant.

**Fig 1:**
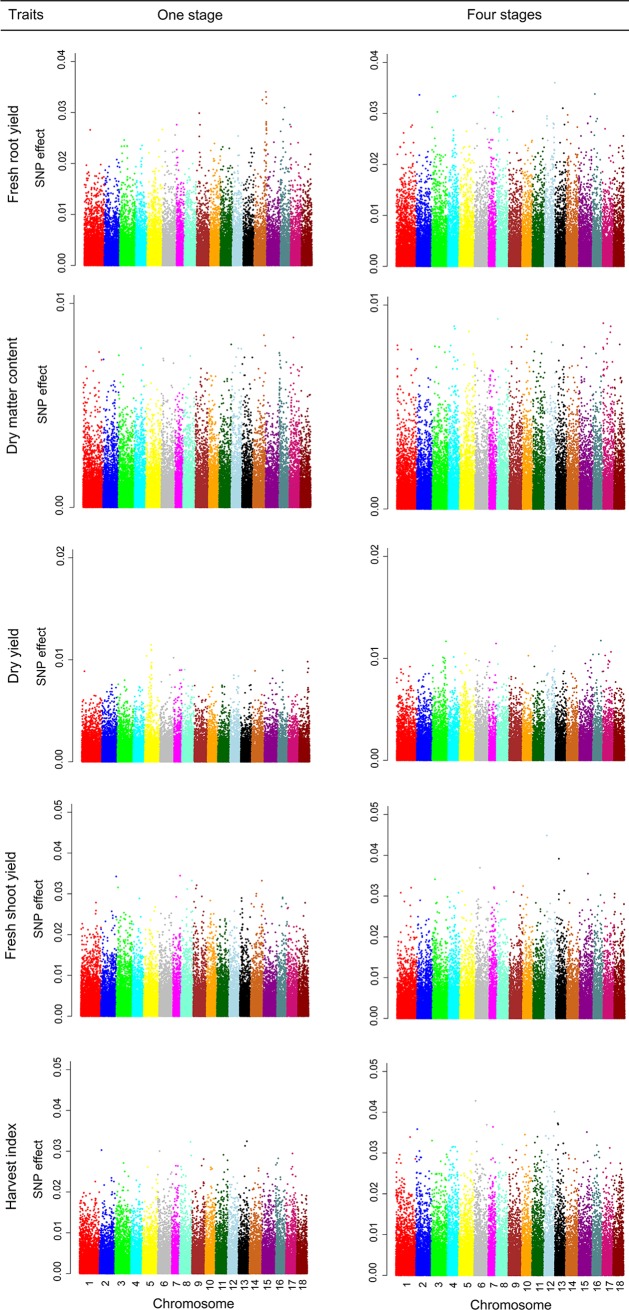
SNP effects for the traits FRY (fresh root yield (t ha^-1^)), DMC (dry matter content (%)), DY (dry yield (t ha^-1^)), FSY (fresh shoot yield (t ha^-1^)) and HI (harvest index (%)), considering only one and four stages of evaluation.

The SNP effects for the five traits, considering one and four stages of evaluation, are shown in Fig 1. Comparing the one-stage with the four-stage evaluation graphs, one can see differences in terms of which chromosome the higher-effect SNPs were located on and also in the magnitude of the effects resulting from the addition of extra information from one stage to four stages of evaluation. These differences could be explained by the fact that in the analyses that included only one stage, the clones were evaluated at four locations, while in the analyses that included the four stages of selection, the clones were evaluated in ten locations and the amount of individual information doubled (Table 2).

### Comparisons

If the results of the one-stage and four-stage evaluations are comparable, it would verify the possibility of using the results from genomic analysis to select clones at the initial stage of a cassava breeding program. To perform this comparison, we compared the rankings of the top 10 clones based on the one-stage genomic analysis (aiming at a reduction of the selection interval) and the four-stage pedigree analysis (complete data set) for each trait as well as for the selection index. We also calculated the correlations between GEBVs (one-stage analysis) and EBVs (four-stage analysis), which were high for all traits, ranging from 0.82 (FRY and DY) to 0.87 (FSY).

For FRY, all clones in the top 10 ranking based on the one-stage genomic analysis were from the cross of Fécula Branca and BRS Formosa, indicating that their progeny represent an important full-siblings family for this trait. There was a good correspondence between the GEBVs from the one-stage genomic analysis and the EBVs from the four-stage pedigree analysis, with a correlation of 0.82 (S1 Table). Many of the clones in the two top-10 rankings coincided (eight out of ten, from the ‘Fécula Branca x BRS Formosa’ crossing). In the top 10 based on the four-stage pedigree analysis, a clone from the cross between BGM-1683 and Fécula Branca also stood out.

There was a good correspondence between GEBVs and EBVs for DMC (correlation of 0.83), and the one-stage and four-stage top 10 lists shared five clones. Progeny from the cross between Fécula Branca and BRS Formosa also stood out for this trait, as well as crossings between the clones Equador72 and BGM-0728, Equador72 and BGM-1124, Cascuda and BGM-0728, BGM-1662 and Fécula Branca and the BRS Mulatinha self-crossing (S2 Table).

For DY, all of the clones in the rankings originated from the cross between Fécula Branca and BRS Formosa, and there was a good correspondence between the GEBVs and the EBVs (correlation of 0.82) (S3 Table). There was a high coincidence between the clones presented in the top 10 rankings for the traits FRY and DY (nine out of ten clones), which was already expected since those traits were highly correlated (0.99).

Fresh shoot yield was the trait that presented the highest amplitude in the top 10 ranking list. The amplitude of GEBVs (one-stage genomic analysis) for the top 10 clones was 20.90; for EBVs (four-stage pedigree analysis), the amplitude was 8.62. This trait also presented a high correlation between the GEBVs and the EBVs (0.87) (S4 Table).

The same pattern was observed for HI, which presented a correlation of 0.84 between GEBVs and EBVs. Moreover, for this trait, all clones in both rankings were from the crossing of Fécula Branca and BRS Formosa (S5 Table).

Assuming that it is a good idea to select clones based on their GEBVs obtained from the one-stage genotypic analysis, the genetic gains from selecting the top 10% of clones (29 clones) were calculated for each trait. The genetic gains were as follows: 15.91% for FRY, 5.15% for DMC, 14.76% for DY, 43.11% for FSY, and 8.47% for HI.

For the selection index, the correlation between GEBVs and EBVs was 0.83 (Table 9). Comparing the top 10% of the clones based on the rankings from these analyses, it was observed that there was a coincidence of 72.41% (21 out of 29 clones). Several clones from Fécula Branca × BRS Formosa crosses presented good performance, as previously noted for individual traits. However, clones from a considerable number of other crosses were also highlighted, such as Equador 72 × Eucalipto, Equador 72 × BGM-0728, BGM-1683 × Fécula Branca, and BRS Formosa × BRS Jari. The genetic gains made by selecting the top 10% of clones based on SI (calculated from the GEBVs of the one-stage analysis) were estimated as follows: 15.13% for FRY, 0.39% for DMC, 14.38% for DY, 24.97% for FSY, and 1.85% for HI.

**Table 9:** Comparison of top 10% rankings between selection index (SI) values based on genomic estimated breeding values (one evaluation stage) or on estimated breeding values (four stages).

Correlation between GEBVs (one stage genomic analysis) and EBVs (four stages pedigree analysis) = 0.83							
Genomic analysis–One stage				Pedigree analysis–Four stages			
Clone	GEBV	Male genitor	Female genitor	Clone	EBV	Male genitor	Female genitor
2012_108_043	1459.09	Fécula Branca	BRS Formosa	2012_108_043	1442.70	Fécula Branca	BRS Formosa
2012_108_208	1402.01	Fécula Branca	BRS Formosa	2012_108_208	1423.61	Fécula Branca	BRS Formosa
2012_108_108	1333.69	Fécula Branca	BRS Formosa	2012_108_108	1413.39	Fécula Branca	BRS Formosa
2012_108_215	1310.99	Fécula Branca	BRS Formosa	2012_108_060	1341.44	Fécula Branca	BRS Formosa
2012_108_060	1308.46	Fécula Branca	BRS Formosa	2012_108_188	1338.13	Fécula Branca	BRS Formosa
2012_108_155	1285.94	Fécula Branca	BRS Formosa	2012_108_155	1310.21	Fécula Branca	BRS Formosa
2012_108_188	1285.12	Fécula Branca	BRS Formosa	2012_108_046	1283.49	Fécula Branca	BRS Formosa
2012_108_046	1267.61	Fécula Branca	BRS Formosa	2014_013_28	1239.57	Equador72	BGM-0728
2012_108_035	1254.20	Fécula Branca	BRS Formosa	2012_108_036	1227.49	Fécula Branca	BRS Formosa
2012_108_036	1250.92	Fécula Branca	BRS Formosa	2012_108_143	1226.44	Fécula Branca	BRS Formosa
BRS Kiriris	1238.41	-	-	2014_018_39	1224.53	Equador72	Eucalipto
2014_018_06	1207.62	Equador72	Eucalipto	2014_013_07	1220.31	Equador72	BGM-0728
2012_108_185	1201.35	Fécula Branca	BRS Formosa	2012_108_185	1218.95	Fécula Branca	BRS Formosa
2012_108_143	1198.61	Fécula Branca	BRS Formosa	2012_108_035	1216.20	Fécula Branca	BRS Formosa
2014_001_34	1192.00	BGM-1332	Fécula Branca	2012_108_041	1205.53	Fécula Branca	BRS Formosa
2014_006_48	1191.36	BGM-1683	Fécula Branca	2014_006_33	1202.38	BGM-1683	Fécula Branca
2012_108_041	1182.19	Fécula Branca	BRS Formosa	2012_108_186	1197.24	Fécula Branca	BRS Formosa
2012_108_129	1178.80	Fécula Branca	BRS Formosa	2012_108_215	1196.28	Fécula Branca	BRS Formosa
2012_108_050	1171.34	Fécula Branca	BRS Formosa	2012_108_050	1187.90	Fécula Branca	BRS Formosa
2012_108_062	1170.54	Fécula Branca	BRS Formosa	2014_006_48	1184.81	BGM-1683	Fécula Branca
2011_34_41	1160.19	Irará	BRS Kiriris	2014_025_42	1184.05	BGM-1662	Fécula Branca
2012_108_038	1159.94	Fécula Branca	BRS Formosa	2014_013_30	1183.75	Equador72	BGM-0728
2012_108_071	1157.50	Fécula Branca	BRS Formosa	2012_108_062	1176.65	Fécula Branca	BRS Formosa
2012_107_002	1156.17	BRS Formosa	BRS Jari	2012_108_129	1174.30	Fécula Branca	BRS Formosa
2014_013_28	1153.43	Equador72	BGM-0728	BRS Kiriris	1166.00	-	-
2012_108_092	1148.86	Fécula Branca	BRS Formosa	2012_108_071	1163.47	Fécula Branca	BRS Formosa
2014_001_08	1148.75	BGM-1332	Fécula Branca	2012_108_092	1161.13	Fécula Branca	BRS Formosa
2011_24_156	1147.67	Lagoa	Mani-Branca	2012_108_132	1160.68	Fécula Branca	BRS Formosa
2012_108_206	1145.19	Fécula Branca	BRS Formosa	2014_012_13	1155.49	Cascuda	BGM-0728

## Discussion

Cassava breeding is usually based on a recurrent phenotypic selection with several stages. For some traits, the improvement rate in cassava is slow due to a combination of several problems related to the biology of cassava. Genomic selection could be one promising approach to reduce the selection interval by predicting the agronomic potential of clones earlier in the process [16, 15, 10].

In our study, we evaluated 290 cassava clones from biparental crosses, which were genotyped and phenotyped for productive traits. Our major objective was to characterize the set of clones, determining genetic parameters relying on pedigree and marker information, as well as to compare the results of one and four selection stages to verify the feasibility of genomic selection at the program’s early stage. We found substantial genetic variation among the 290 cassava clones for all traits evaluated. Moreover, they showed intermediate to high heritability, and consequently, good progress can be made in selecting clones for those traits. The majority of traits presented significant genotype × location interaction effects (*p* ≤ 0.01), with the exception of DMC (one-stage pedigree analysis and one- and four-stage genomic analyses) and HI (one-stage pedigree analysis).

The coefficient of determination of the interaction represents how much of the total variation is explained by the genotype × location interaction. According to our results, DMC and HI appear less impacted by genotype × location interactions and their impacts than the other traits (FRY, DY and FSY).

FRY and DMC are important agronomic traits for cassava, and the mean trait values found in the present work were consistent with the results of [28]. They evaluated 627 genotypes in a clonal trial, and their estimates for FRY ranged from 9.64 to 63.66 t ha^-1^ (mean of 34.37 t ha^-1^) and for DMC from 20.65 to 41.25% (mean of 33.47%). In the study by [6], they performed successive stages of phenotypic assessment of cassava adapted to the sub-humid environment, and the observed means for FRY, DMC and HI were 24.00 t ha^-1^, 33.57% and 58%, respectively. In the present study, when only the one-stage pedigree analysis (CET) was assessed, we found means of 29.24 t ha^-1^, 33.49% and 70.62%, for FRY, DMC and HI, respectively (Table 4).

Dry matter content is usually what determines the price paid to cassava producers since it is directly related to the starch yield and, consequently, its several derivative products [29, 30]. Despite its importance for starch yield (SY), the correlations between DMC and DY (which is a redundant trait for SY since the correlation between SY and DY is 0.99 (data not shown)) in the present work were low, ranging from 0.04 to 0.11 (Tables 5 and 8), which differs from the values reported by [30], where the genetic and phenotypic correlations for DMC and SY were 0.36 and 0.42, respectively.

Harvest index is commonly considered an indirect cassava yield indicator based on reports of high correlation between HI in single row trials and FRY performance in replicated plots in multi-location trials [31, 3]. In our work, the correlations between HI and FRY were moderate, ranging from 0.26 to 0.41 (Tables 5 and 8). HI and FSY are negatively correlated traits. It is important to be aware that plants with high HI and low FSY, even with high-yielding roots, are undesirable because they produce little propagating material. In this case, a high HI may not reflect a high-yielding root, but a low production of fresh shoot yield. It is important to seek a balance between the production of roots and shoot [32].

The total dataset consisted of clones from biparental crosses and commercial clones (used as parents and controls); therefore, there were half-sibling and full-sibling populations (clones sharing one or two parents, respectively). According to [15], using GS to achieve a shorter interval cycle is predicted to be favorable within full-sibling families because biparental populations have high linkage disequilibrium (LD) between marker alleles and trait alleles with no group structure.

All traits presented moderate to high heritability, which shows great potential for obtaining genetic gains. Traits with higher heritability tend to show higher accuracy, but this was not the case in some situations for the present work. For example, in one-stage genomic analysis, FSY and HI presented the highest accuracies (0.69 and 0.71, respectively), but the heritability of DMC (0.66) was higher than those of FSY and HI (0.45 and 0.47, respectively; Table 7). As reported by [33], those discrepancies can presumably be caused by the proximity of SNP markers to the QTL (quantitative trait loci) and the genetic architecture. In pedigree and genotypic analyses, DMC was the trait that presented the highest heritability, which might be related to the fact that DMC was the only trait that did not present a significant (*p ≤* 0.01) genotype × location interaction effect (Table 6). According to [33], the genotype × location effect and the relatedness among genotypes affect the precision of genomic selection estimates.

Using only the most informative SNPs, [16] observed an increase in the accuracy for the traits FRY, DMC and SY. In their work, 358 cassava accession were evaluated using 390 marker SNPs, and broad heritabilities (obtained by REML analysis of phenotypic data) of 0.72, 0.67 and 0.69, respectively, were found. For FRY, DMC and SY, they obtained accuracies of 0.31, 0.20 and 0.33, respectively. When they selected markers, the accuracies were 0.76, 0.67 and 0.77 for FRY, DMC and SY, respectively, with predictive abilities of 0.38, 0.30 and 0.39. In the present work, in our four-stage genomic analysis, we observed predictive abilities for FRY, DMC and DY of 0.50, 0.59 and 0.51, respectively (Table 7), without selecting markers, taking into consideration 51,259 SNPs. The heritabilities for FRY, DMC, FSY and HI were considerably higher (ranging from 0.46 to 0.69; four-stage, Table 7) than those reported by [33], which ranged from 0.11 to 0.28. [33] adopted three different cross-validation (CV) approaches: CV without close relatives, random fivefold CV and CV with close relatives. Accuracies varied from 0.46 to 0.48 for DMC, from 0.43 to 0.48 for HI, from 0.36 to 0.41 for FRY, and from 0.23 to 0.30 for FSY; the lowest accuracy value was obtained using CV without close relatives, and the highest was obtained by random fivefold CV. The genomic accuracies that we have presented in this study varied from 0.60–0.70, 0.56–0.71, 0.61–0.69 and 0.68–0.72 for DMC, HI, FRY and FSY, respectively. It is possible that the values presented here were higher because we adopted a random CV approach and because we had a considerable number of related clones.

[10] analyzed data from three African cassava research institutions and assessed the accuracy of seven prediction models for seven traits. Those authors reported that some trait-dataset combinations exhibited better predictive accuracies than others. For example, for DMC, the estimated accuracies varied from 0.27 to 0.66 across the evaluated breeding programs. According to [34] and [15], when a large number of loci controls the trait, genomic accuracy depends on several factors, among them the training population size, trait heritability and genetic diversity and its relationship with the test population.

The comparisons between the top 10 clone rankings of the one-stage genomic analysis and the four-stage pedigree analysis provided insights about the feasibility of using the clonal evaluation trials’ GEBVs for early selection. This approach would reduce the selection interval and consequently minimize the extensive, costly and time-consuming phenotypic evaluations that are usually conducted in a traditional cassava breeding program [35]. Among the traits, the correlation between GEBVs (one-stage genomic analysis) and EBVs (four-stage pedigree analysis) ranged from 0.82 to 0.87, and between 50 and 90% of their top 10 rankings coincided. The clones that originated from the ‘Fécula Branca x BRS Formosa’ crosses stood out for all of the traits as well as for the selection index. This indicates that this specific combination is favorable for the improvement of cassava productive traits. The feasibility of using genomic selection at the early stages of evaluation is further supported by the fact that the accuracy estimates obtained for the genomic analysis that included only the clonal evaluation trial (one stage) ranged from 0.60 (DMC) to 0.71 (HI). The harvest index, which was the trait that presented the highest accuracy in the one-stage genomic analysis, is an important trait for cassava breeding programs as it is usually used as an indirect indicator of cassava yield.

The genetic gains, calculated separately for each trait (based on the selection of the top 10% of clones by GEBVs obtained by means of the one-stage genotypic analysis), represent great perspectives for cassava breeding (genetic gains of 15.91% for FRY, 5.15% for DMC, 14.76% for DY, 43.11% for FSY, and 8.47% for HI, respectively). [30] calculated the genetic gains for DMC and SY, selecting 30 out of 471 clones. They obtained genetic gains of 10.75 and 5.50% for DMC and 74.62 and 49.95% for SY when comparing the overall averages of the experiments and controls, respectively. On the other hand, the genetic gains when selecting the best 10% of clones based on the proposed selection index (calculated with GEBVs from the one-stage genotypic analysis) were as follows: 15.13% for FRY, 0.39% for DMC, 14.38% for DY, 24.97% for FSY, and 1.85% for HI. Although the selection gains were lower when based on the selection index compared to the selection by trait individually, with the selection index, it is possible to improve key traits simultaneously [6].

It is worth mentioning that although many of the clones with high selection index values come from the Fécula Branca × BRS Formosa crossing, many others came from other crossings. According to [7], this variability weakens the identity of families and supports the idea that outstanding hybrids can be obtained from essentially every family. This idea arises from the large within-family segregations (due to the fact that progenitors are generally heterozygous) that cassava breeders observe in their evaluation fields.

Traditional assessments based solely on phenotypic information are still very effective and work well for cassava breeding since most of the traits have high heritability. However, here, we wanted to present genomic selection as an option and a great tool to obtain accurate estimates by assessing clones’ genomic estimated breeding value earlier in the cassava breeding cycle, thus saving resources such as time, planting area, and direct and indirect costs. The evaluated clones that stood out in the present work could be new varieties to be released in the future and/or could be used as a germplasm source for the development of new varieties with high allele frequencies for starch-related productive traits.

## Conclusions

The heritability and accuracy values obtained from genomic and pedigree analyses were moderate to high for important traits related to starch production. The results indicate excellent potential for breeding and genomic selection in these cassava populations aimed at increasing starch production for commercial use. In addition, they indicate an optimal potential for selection at early stages of a cassava breeding program (CET), since the correlations between the GEBVs of one-stage genomic analysis and the EBVs of the four-stage pedigree analysis were high for all traits. Moreover, the GEBV-based and EBV-based rankings of the top 10 best clones overlapped by 5 to 9 clones for individual traits, and the rankings of the 10% best clones exhibited 72% overlap for the selection index. The estimated genetic gains obtained by using the 10% best clones identified by the selection index with the GEBVs from the one-stage genomic analysis were 15.13%, 0.39%, 14.38%, 24.95% and 1.84% for FRY, DMC, DY, FSY and HI, respectively. Thus, genomic selection enables effective early selection in the first stage of clonal evaluation.

## Supporting information

S1 Table Comparison of top 10 rankings based on genomic estimated breeding value (one evaluation stage) or on estimated breeding value (four stages) for fresh root yield (FRY, in t ha^-1^). (DOCX)

S2 Table Comparison of top 10 rankings based on genomic estimated breeding value (one evaluation stage) or on estimated breeding value (four stages) for dry matter content (DMC, in %). (DOCX)

S3 Table Comparison of top 10 rankings based on genomic estimated breeding value (one evaluation stage) or on estimated breeding value (four stages) for dry yield (DY, in t ha^-1^). (DOCX)

S4 Table Comparison of top 10 rankings based on genomic estimated breeding value (one evaluation stage) or on estimated breeding value (four stages) for fresh shoot yield (FSY, in t ha^-1^). (DOCX)

S5 Table Comparison of top 10 rankings based on genomic estimated breeding value (one evaluation stage) or on estimated breeding value (four stages) for harvest index (HI, in %). (DOCX)

S1 Dataset i) estimated breeding values (EBVs) and genomic estimated breeding values (GEBVs) based on the pedigree and genomic analysis, respectively, for the different breeding stages were analyzed as follows: one stage (Clonal evaluation trial—CET), two stages (CET and preliminary yield trial—PYT), three stages (CET, PYT and advanced yield trial—AYT) and four stages (CET, PYT, AYT and uniform yield trial—UYT); ii) phenotypic raw data; iii) SNPs effects for the two breeding atges (one and four), and iv) SNPs location on the cassava chromosome.
(RAR)

## References

[1] FAO. Cassava for food and energy security. FAO Newsroom. 2008. http://www.fao.org/newsroom/en/news/2008/1000899/index.html. Accessed in 29 Mai 2018.

[2] Ceballos H, Kawuki RS, Gracen VE, Yencho GC, Hershey CH. Conventional breeding, marker assisted selection, genomic selection and inbreeding in clonally propagated crops: a case study for cassava. Theoretical and Applied Genetics. 2015; 9:1647 10.1007/s00122-015-2555-4 PMC4540783 26093610

[3] Karlström A, Calle F, Salazar S, Morante N, Dufour D, Ceballos H. Biological Implications in Cassava for the Production of Amylose-Free Starch: Impact on Root Yield and Related Traits. Frontiers in Plant Science. 2016; 7: 604 10.3389/fpls.2016.00604 27242813 PMC4873506

[4] El-Sharkawy M.A. Cassava Biology and Physiology. Plant Molecular Biology. 2004; 56, 481–501. 15669146 10.1007/s11103-005-2270-7

[5] Saithong T, Saerue S, Kalapanilak S, Sojikul P, Narangajavana J, Bhumiratana S. Gene Co-Expression Analysis Inferring the Crosstalk of Ethylene and Gibberellin in Modulating the Transcriptional Acclimation of Cassava Root Growth in Different Seasons. Plos One. 2015; 10(9):e0137602 10.1371/journal.pone.0137602 26366737 PMC4569563

[6] Barandica OJ, Pérez JC, Lenis JI, Calle F, Morante N, Pino L, Hershey C.H, Ceballos H. Cassava Breeding II: Phenotyphic Correlations through the Different Stages of Selection. Frontiers in Plant Science. 2016; 7:1649 10.3389/fpls.2016.01649 28018365 PMC5156711

[7] Ceballos H, Pérez JC, Barandica OJ, Lenis JI, Morante N, Calle F, Pino L, Hershey CH. Cassava Breeding I: The Value of Breeding Value. Frontiers in Plant Science. 2016; 7:1227 10.3389/fpls.2016.01227 27621734 PMC5003041

[8] Kayondo SI, Carpio DPD, Lozano R, Ozimati A, Wolfe M, Baguma Y, Gracen V, Samuel O, Ferguson M, Kawuki R, Jannink J. Genome-wide association mapping and genomic prediction unravels CBSD resistance in a 2 Manihot esculenta breeding population. Scientific Reports. 2018; 8:1549 10.1038/s41598-018-19696-1 29367617 PMC5784162

[9] Jennings DL and Iglesias CA. Breeding for crop improvement in Cassava: Biology, Production and Utilization Wallingford: CABI Publishing; 2002.

[10] Wolfe MD, Carpio DPD, Alabi O, Ezenwaka LC, Ikeogu UN, Kayondo IS, Lozano R, Okeke UG, Ozimati AA, Williams E, Egesi C, Kawuki RS, Kulakow P, Rabbi IY, Jannink J. Prospects for Genomic Selection in Cassava Breeding. The Plant Genome. 2017; 10:1–19. 10.3835/plantgenome2017.03.0015 PMC7822052 29293806

[11] Sanginga N. Root and Tuber Crops (Cassava, Yam, Potato and Sweet Potato). Feeding Africa. 2015. https://www.afdb.org/fileadmin/uploads/afdb/Documents/Events/DakAgri2015/Root_and_Tuber_Crops__Cassava__Yam__Potato_and_Sweet_Potato_.pdf. Accessed in 27 Sept 2018.

[12] Sakurai T, Mochida K, Yoshida T, Akiyama K, Ishitani M, Seki M. et al Genome-Wide Discovery and Information Resource Development of DNA Polymorphisms in Cassava. Plos One. 2013; 8(9): e74056 10.1371/journal.pone.0074056 24040164 PMC3770675

[13] Prochnik S, Marri PR, Desany B, Rabinowicz PD, Kodira C, Mohiuddin M, Rodriguez F, Fauquet C, Tohme J, Harkins T, Rokhsar DS, Rounsley S. The Cassava Genome: Current Progress, Future Directions. Tropical Plant Biology. 2012; 5:88–94. 10.1007/s12042-011-9088-z 22523606 PMC3322327

[14] Meuwissen THE, Hayes BJ, Goddard ME. Prediction of total genetic value using genome-wide dense marker maps. Genetics. 2001; 157:1819 11290733 10.1093/genetics/157.4.1819 PMC1461589

[15] Crossa J, Pérez-Rodríguez P, Cuevas J, Montesinos-López O, Jarquín D, de los Campos G, Burgueño J, González-Camacho JM, Pérez-Elizalde S, Beyene Y, Dreisigacker S, Singh R, Zhang X, Gowda M, Roorkiwal M, Rutkoski J, Varshney RK. Genomic Selection in Plant Breeding: Methods, Model, and Perspectives. Trends in Plant Science. 2017; 22(11):961–975. 10.1016/j.tplants.2017.08.011 28965742

[16] Oliveira EJ, Resende MDV, Santos VS, Ferreira CF, Oliveira GAF, da Silva MS, de Oliveira LA, Aguilar-Vildoso CI. Genome-wide selection in cassava. Euphytica. 2012; 187:263.

[17] Souza LS, Farias AR, Mattos PLP, Fukuda WMG. Aspectos socioeconômicos e agronômicos da mandioca. Embrapa Mandioca e Fruticultura Tropical; 2006.

[18] Kawano K, Fukuda WMG, Cenpukdee U. Genetic and environmental effects on dry matter content of cassava root. Crop Science. 1987; 27:69–74.

[19] Doyle JJ and Doyle JL. Isolation of plant DNA from fresh tissue. Focus. 1990; 12, 13–15.

[20] Elshire RJ, Glaubitz JC, Sun Q, Poland JA, Kawamoto K, Buckler ES, Mitchell SE. A robust, simple genotyping-by-sequencing (GBS) approach for high diversity species. Plos One. 2011; 6, 1–10. 10.1371/journal.pone.0019379 PMC3087801 21573248

[21] Resende MDV, Azevedo CF, Silva FF, Gois IB. Atualidades da biometria no melhoramento de plantas perenes In: Desafios biométricos no melhoramento genético. eDOC Brasil; 2017 pp 67–84.

[22] Poland J, Endelman J, Dawson J, Rutkoski J, Wu S, Manes Y, Dreisigacker S, Crossa J, Sánchez-Villeda H, Sorrells M, Jannick, J. Genomic Selection in Wheat Breeding using Genotyping-by-Sequencing. The Plant Genome. 2012; 5:103:113.

[23] VanRaden PM. Efficient methods to compute genomic predictions. Journal of Dairy Science. 2008; 91:4414 10.3168/jds.2007-0980 18946147

[24] Resende MDV. Selegen-REML/BLUP: Sistema estatístico e seleção genética computadorizada via modelos lineares mistos. Embrapa Florestas; 2007.

[25] Covarrubias-Pazaran G. Genome assisted prediction of quantitative traits using the R package sommer. Plos One. 2016; 11(6):1–15. 10.1371/journal.pone.0156744 27271781 PMC4894563

[26] R Core Team. R: A language and environment for statistical computing R Foundation for Statistical Computing, Vienna, Austria Available in: http://www.R-project.org.

[27] Resende MDV, Duarte JB. Precisão e controle de qualidade em experimentos de avaliação de cultivares. Pesquisa Agropecuária Tropical. 2007; 37(3):182–194.

[28] Ojulong H, Labuschangne MT, Fregene M, Herselman L. A cassava clonal evaluation trial based on a new cassava breeding scheme. Euphytica. 2008; 160:119–129.

[29] Carvalho PRN, Mezette TF, Valle TL, Carvalho CRL, Feltran JC. Avaliação da exatidão, precisão e robustez do método de análise do teor de matéria seca de mandioca (Manihot esculenta, Crantz) por meio da determinação do peso específico (balança hidrostática). Revista raízes e amido tropical. 2007; 3:1–4.

[30] Oliveira EJ, Santana FA, Oliveira LA, Santos VS. Genetic parameters and prediction of genotypic values for root quality traits in cassava using REML/BLUP. Genetics and Molecular Research. 2014; 13 (3): 6683–6700. 10.4238/2014.August.28.13 25177949

[31] Kawano K, Daza P, Amaya A, Ríos M, Gonçalvez MF. Evaluation of cassava germplasm for productivity. Crop Science. 1978; 18:377–380.

[32] Farias ARN, Souza LS, Mattos PLP, Fukuda WMG. Aspectos Socioeconômicos e Agronômicos da Mandioca. Embrapa Mandioca e Fruticultura; 2006.

[33] Ly D, Hamblin M, Rabbi I, Melaku G, Bakare M, Gauch HG Jr, Okechukwu R, Dixon AGO, Kulakow P, Jannick J. Relatedness and Genotype x Environment Interaction Affect Prediction Accuracies in Genomic Selection: A Study in Cassava. Crop Science. 2013; 53:1312–1325.

[34] Resende MDV. Genética Quantitativa e de Populações. Editora Suprema; 2015.

[35] Brito AC, Oliveira SAS, Oliveira EJ. Genome-wide association study for resistance to cassava root rot. Journal of Agricultural Science. 2017; 155:1424–1441.

