# Supplementary material for: Genomic selection for productive traits in biparental cassava breeding populations
Source: PLoS One. 2019 Jul 25;14(7):e0220245. doi: 10.1371/journal.pone.0220245 (PMC6658084; doi:10.1371/journal.pone.0220245)
Supplement: S1 Table — (DOCX) [file pone.0220245.s001.docx]

**S1 Table. Comparison of top 10 rankings based on genomic estimated breeding value (one evaluation stage) or on estimated breeding value (four stages) for fresh root yield (FRY, in t ha^-1^).**

| Correlation between GEBVs (one stage genomic analysis) and EBVs (four stages pedigree analysis) = 0.82 | | | | | | | |
| --- | --- | --- | --- | --- | --- | --- | --- |
| Genomic analysis – One stage | | | | Pedigree analysis – Four stages | | | |
| Clone | GEBV | Male genitor | Female genitor | Clone | EBV | Male genitor | Female genitor |
| 2012_108_043 | 56.52 | Fécula Branca | BRS Formosa | 2012_108_043 | 55.55 | Fécula Branca | BRS Formosa |
| 2012_108_208 | 53.67 |  |  | 2012_108_208 | 53.75 |  |  |
| 2012_108_060 | 48.57 |  |  | 2012_108_108 | 52.34 |  |  |
| 2012_108_108 | 48.07 |  |  | 2012_108_060 | 50.88 |  |  |
| 2012_108_046 | 47.64 |  |  | 2012_108_188 | 48.98 |  |  |
| 2012_108_215 | 46.32 |  |  | 2012_108_046 | 48.48 |  |  |
| 2012_108_188 | 45.97 |  |  | 2012_108_143 | 45.21 |  |  |
| 2012_108_035 | 44.95 |  |  | 2012_108_155 | 43.36 |  |  |
| 2012_108_143 | 43.12 |  |  | 2012_108_035 | 42.68 |  |  |
| 2012_108_036 | 43.07 |  |  | 2014_006_33 | 42.45 | BGM-1683 | Fécula Branca |
