# Supplementary material for: Genomic selection for productive traits in biparental cassava breeding populations
Source: PLoS One. 2019 Jul 25;14(7):e0220245. doi: 10.1371/journal.pone.0220245 (PMC6658084; doi:10.1371/journal.pone.0220245)
Supplement: S2 Table — (DOCX) [file pone.0220245.s002.docx]

**S2 Table. Comparison of top 10 rankings based on genomic estimated breeding value (one evaluation stage) or on estimated breeding value (four stages) for dry matter content (DMC, in %).**

| Correlation between GEBVs (One stage genomic analysis) and EBVs (Four stages pedigree analysis) = 0.83 | | | | | | | |
| --- | --- | --- | --- | --- | --- | --- | --- |
| Genomic analysis – One stage | | | | Pedigree analysis – Four stages | | | |
| Clone | GEBV | Male genitor | Female genitor | Clone | EBV | Male genitor | Female genitor |
| 2014_013_41 | 38.32 | Equador72 | BGM-0728 | 2012_108_155 | 37.21 | Fécula Branca | BRS Formosa |
| 2014_008_19 | 38.04 | Equador72 | BGM-1124 | 2012_108_125 | 36.90 | Fécula Branca | BRS Formosa |
| 2012_108_155 | 37.38 | Fécula Branca | BRS Formosa | 2014_008_19 | 36.82 | Equador72 | BGM-1124 |
| 2014_012_13 | 36.68 | Cascuda | BGM-0728 | 2012_108_049 | 36.68 | Fécula Branca | BRS Formosa |
| 2012_108_206 | 36.45 | Fécula Branca | BRS Formosa | 2012_108_061 | 36.57 | Fécula Branca | BRS Formosa |
| 2014_013_24 | 36.44 | Equador72 | BGM-0728 | 2014_025_42 | 36.47 | BGM-1662 | Fécula Branca |
| 2012_108_030 | 36.34 | Fécula Branca | BRS Formosa | 2012_108_030 | 36.43 | Fécula Branca | BRS Formosa |
| 2011_52_01 | 36.33 | BRS Mulatinha | BRS Mulatinha | 2012_108_098 | 36.39 | Fécula Branca | BRS Formosa |
| 2014_008_11 | 36.32 | Equador72 | BGM-1124 | 2012_108_206 | 36.32 | Fécula Branca | BRS Formosa |
| 2012_108_125 | 36.25 | Fécula Branca | BRS Formosa | 2012_108_190 | 36.20 | Fécula Branca | BRS Formosa |
