# Supplementary material for: Genomic selection for productive traits in biparental cassava breeding populations
Source: PLoS One. 2019 Jul 25;14(7):e0220245. doi: 10.1371/journal.pone.0220245 (PMC6658084; doi:10.1371/journal.pone.0220245)
Supplement: S3 Table — (DOCX) [file pone.0220245.s003.docx]

**S3 Table. Comparison of top 10 rankings based on genomic estimated breeding value (one evaluation stage) or on estimated breeding value (four stages) for dry yield (DY, in t ha^-1^).**

| Correlation between GEBVs (one stage genomic analysis) and EBVs (four stages pedigree analysis) = 0.82 | | | | | | | |
| --- | --- | --- | --- | --- | --- | --- | --- |
| Genomic analysis – One stage | | | | Pedigree analysis – Four stages | | | |
| Clone | GEBV | Male genitor | Female genitor | Clone | EBV | Male genitor | Female genitor |
| 2012_108_043 | 18.77 | Fécula Branca | BRS Formosa | 2012_108_043 | 18.49 | Fécula Branca | BRS Formosa |
| 2012_108_208 | 17.54 |  |  | 2012_108_108 | 17.72 |  |  |
| 2012_108_215 | 16.39 |  |  | 2012_108_208 | 17.51 |  |  |
| 2012_108_108 | 16.00 |  |  | 2012_108_060 | 16.43 |  |  |
| 2012_108_060 | 15.62 |  |  | 2012_108_188 | 15.94 |  |  |
| 2012_108_035 | 15.50 |  |  | 2012_108_155 | 15.81 |  |  |
| 2012_108_155 | 15.17 |  |  | 2012_108_046 | 15.46 |  |  |
| 2012_108_046 | 14.98 |  |  | 2012_108_035 | 14.88 |  |  |
| 2012_108_188 | 14.95 |  |  | 2012_108_036 | 14.45 |  |  |
| 2012_108_036 | 14.67 |  |  | 2012_108_143 | 14.27 |  |  |
