# Supplementary material for: Genomic selection for productive traits in biparental cassava breeding populations
Source: PLoS One. 2019 Jul 25;14(7):e0220245. doi: 10.1371/journal.pone.0220245 (PMC6658084; doi:10.1371/journal.pone.0220245)
Supplement: S4 Table — (DOCX) [file pone.0220245.s004.docx]

**S4 Table. Comparison of top 10 rankings based on genomic estimated breeding value (one evaluation stage) or on estimated breeding value (four stages) for fresh shoot yield (FSY, in t ha^-1^).**

| Correlation between GEBVs (one stage genomic analysis) and EBVs (four stages pedigree analysis) = 0.87 | | | | | | | |
| --- | --- | --- | --- | --- | --- | --- | --- |
| Genomic analysis – One stage | | | | Pedigree analysis – Four stages | | | |
| Clone | GEBV | Male genitor | Female genitor | Clone | EBV | Male genitor | Female genitor |
| 2014_018_06 | 49.60 | Equador72 | Eucalipto | 2012_108_185 | 38.43 | Fécula Branca | BRS Formosa |
| 2012_108_222 | 37.21 | Fécula Branca | BRS Formosa | 2012_108_208 | 34.85 | Fécula Branca | BRS Formosa |
| 2014_013_28 | 36.02 | Equador72 | BGM-0728 | 2014_013_28 | 34.81 | Equador72 | BGM-0728 |
| 2012_108_185 | 34.42 | Fécula Branca | BRS Formosa | 2012_108_222 | 32.18 | Fécula Branca | BRS Formosa |
| 2012_108_224 | 31.46 | Fécula Branca | BRS Formosa | 2014_018_06 | 31.70 | Equador72 | Eucalipto |
| 2014_013_07 | 29.59 | Equador72 | BGM-0728 | 2012_108_188 | 31.68 | Fécula Branca | BRS Formosa |
| 2012_108_208 | 29.42 | Fécula Branca | BRS Formosa | 2012_108_224 | 30.21 | Fécula Branca | BRS Formosa |
| 2012_108_043 | 29.25 | Fécula Branca | BRS Formosa | 2014_025_42 | 30.11 | BGM-1662 | Fécula Branca |
| 2014_013_30 | 29.01 | Equador72 | BGM-0728 | 2014_026_33 | 29.88 | BGM-1683 | Fécula Branca |
| 2012_106_160 | 28.70 | Fécula Branca | BRS Jari | 2012_108_108 | 29.81 | Fécula Branca | BRS Formosa |
