# Supplementary material for: Genomic selection for productive traits in biparental cassava breeding populations
Source: PLoS One. 2019 Jul 25;14(7):e0220245. doi: 10.1371/journal.pone.0220245 (PMC6658084; doi:10.1371/journal.pone.0220245)
Supplement: S5 Table — (DOCX) [file pone.0220245.s005.docx]

**S5 Table. Comparison of top 10 rankings based on genomic estimated breeding value (one evaluation stage) or on estimated breeding value (four stages) for harvest index (HI, in %).**

| Correlation between GEBVs (one stage genomic analysis) and EBVs (four stages pedigree analysis) = 0.84 | | | | | | | |
| --- | --- | --- | --- | --- | --- | --- | --- |
| Genomic analysis – One stage | | | | Pedigree analysis – Four stages | | | |
| Clone | GEBV | Male genitor | Female genitor | Clone | EBV | Male genitor | Female genitor |
| 2012_108_034 | 89.61 | Fécula Branca | BRS Formosa | 2012_108_179 | 88.56 | Fécula Branca | BRS Formosa |
| 2012_108_179 | 88.78 |  |  | 2012_108_021 | 88.24 |  |  |
| 2012_108_205 | 88.69 |  |  | 2012_108_034 | 86.49 |  |  |
| 2012_108_149 | 88.38 |  |  | 2012_108_149 | 86.44 |  |  |
| 2012_108_021 | 88.12 |  |  | 2012_108_205 | 86.25 |  |  |
| 2012_108_144 | 88.00 |  |  | 2012_108_029 | 86.15 |  |  |
| 2012_108_187 | 87.46 |  |  | 2012_108_162 | 86.12 |  |  |
| 2012_108_006 | 87.20 |  |  | 2012_108_143 | 85.14 |  |  |
| 2012_108_035 | 86.92 |  |  | 2012_108_006 | 84.87 |  |  |
| 2012_108_162 | 86.91 |  |  | 2012_108_161 | 84.85 |  |  |
